# Role of Glycerophospholipid Metabolism in Epilepsy

**DOI:** 10.2174/011570159X374966250319050052

**Published:** 2025-04-07

**Authors:** Zijian Li, Zhen Liang, Jing Zhang, Songyan Liu

**Affiliations:** 1 Department of Neurology, China-Japan Union Hospital of Jilin University, Changchun, Jilin Province, 130033, China

**Keywords:** Glycerophospholipids, epilepsy, metabolism, inflammation, oxidative stress, gene mutation, targets

## Abstract

Epilepsy is a prevalent and severe neurological condition characterized by recurring seizures. It impacts over 70 million individuals worldwide, posing a substantial challenge to public health and placing a heavy burden on society. Glycerophospholipids are an essential component of neuronal cell membranes. Their metabolism is strictly regulated, and maintaining their homeostasis is crucial for the optimal function of the nervous system. Research indicates that disruptions in glycerophospholipid metabolism are commonly detected in patients with epilepsy and animal models. However, the precise molecular mechanisms behind these disruptions remain unclear. Existing evidence indicates that neuroinflammation, oxidative stress, genetic mutations, and ion channel dysfunction are crucial factors contributing to glycerophospholipids imbalance and epilepsy. Further, therapeutic interventions targeting these pathological processes, such as regulating neuroinflammation and oxidative stress or restoring the balance of glycerophospholipid metabolism, may provide new avenues for epilepsy treatment. This review aims to provide an in-depth analysis of the potential mechanisms underlying the relationship between glycerophospholipid metabolism disorders and epilepsy, exploring potential therapeutic targets and diagnostic biomarkers.

## 1 INTRODUCTION

Epilepsy, a neurological condition, arises from abnormal simultaneous discharges of neurons and affects around 70 million individuals globally. Its clinical features are paroxysmal, transient, repetitive, and stereotyped [1]. The clinical diagnosis of epilepsy relies heavily on electroencephalography. However, in the absence of electroencephalography, differentiating epilepsy from other neurological disorders is challenging. Despite advancements in anti-seizure medications (ASMs) in recent years, approximately 30% of patients are diagnosed with drug-resistant epilepsy [2]. Therefore, investigating the pathogenesis of epilepsy and exploring relevant biomarkers and new treatment targets remain critical challenges in this field.

Eukaryotic cell membranes are intricate structures composed of a diverse array of lipid molecules and proteins, with phospholipids (PLs) serving as essential components. Glycerophospholipids (GPLs), the predominant PLs in the brain, constitute approximately 20-25% of its dry weight [3]. GPLs consist of glycerol, fatty acids, and phosphate groups. The glycerol molecule is linked to the phosphate and substituent group-X in the sn-3 position. Different GPLs are formed due to different substituent groups-X. The fatty acid chain at the sn-2 position is attached to the glycerol molecule *via* an ester bond, whereas the chain at the sn-1 position may be linked by an ester, ether, or vinyl ether bond. The alkyl chain length typically ranges from 14 to 24 carbon atoms and contains 0-6 double bonds. Fatty acids at the sn-1 position are generally saturated or monounsaturated, while those at the sn-2 position are predominantly polyunsaturated. These numerous combinations of fatty acid chains enable a single eukaryotic cell to contain hundreds of distinct GPL molecules [4]. In the brain, GPLs primarily comprise phosphatidylcholines (PCs), phosphatidylserines (PSs), phosphatidylinositols (PIs), phosphatidylethanolamines (PEs), phosphatidylglycerols (PGs), and cardiolipins (CLs) [5]. The heterogeneity of GPL molecules arises from variations in compositions of fatty acids and head groups, and their distribution within nerve membranes influences their metabolism and physicochemical properties [6]. Nerve membranes are densely populated with receptors, enzymes, and ion channels located on intracellular or extracellular surfaces, interacting dynamically with the phospholipid bilayer. Together, they participate in physiological processes like endocytosis, exocytosis, membrane fusion, signal transduction, and the intake and release of neurotransmitters, directly impacting neuronal function [5, 7, 8]. In addition, GPLs are not only the main component of biological membranes but also have other vital functions. For example, GPLs can be degraded to arachidonic acid (AA) to participate in inflammation or act as precursors of endocannabinoids (eCBs), which play an essential role in neuroprotection [9, 10].

The concentration of GPLs in mammals is usually tightly regulated, which means that even a slight change in the level may lead to harmful consequences. A previous study showed that knocking out the key enzymes responsible for GPL biosynthesis in mice leads to embryonic lethality or severe organ damage [11]. Moreover, reduced levels of PIs, PEs, and PCs have been detected in the brain tissue of individuals with Alzheimer's disease, highlighting a potential link between altered GPL metabolism and neurodegeneration [12, 13]. PLs are also implicated in the metabolism of amyloid-beta, a classic pathological marker of Alzheimer's disease [14]. Boska *et al*. [15] reported that Mg^2+^ and membrane PLs undergo compensatory changes in patients with migraine without neurologic aura, counteracting cortical excitability. In addition, oxidized phospholipids (OxPLs), which are products of oxidative damage to PLs, possess pro-inflammatory properties and are central to the pathogenesis of Parkinson's disease, Alzheimer's disease, and traumatic brain injury [16]. In summary, significant changes in nerve membrane GPL composition are common in neurological disorders, potentially leading to decreased membrane stability, OxPLs accumulation, inflammatory response, and impaired energy metabolism, further promoting the occurrence of nervous system diseases.

Recently, there has been a growing focus on the role of GPL metabolism in epilepsy. As the main component of neuronal cell membranes, disturbances in GPLs are closely associated with epilepsy. Lipid profiles in patients with epilepsy have been widely studied, providing insights into lipid identification and associated metabolic pathways as potential avenues for novel treatment strategies and biomarkers [17, 18]. Although several studies have explored the association between abnormal GPL metabolism and epilepsy, most of these investigations relied on brain tissue specimens. In general, abnormal GPL metabolism is common in both patients with epilepsy and animal models. However, the results of various studies are inconsistent, and the metabolic characteristics of GPLs also differ between brain regions and even within the same brain structure [19-24]. Therefore, it is difficult to interpret the results of existing studies in this context, and currently impossible to reach a uniform conclusion. This review investigates the link between GPL metabolism and epilepsy, aiming to enhance our understanding of epilepsy's pathological mechanisms and to identify potential therapeutic targets and biomarkers.

## 2 ROLE OF GPL METABOLISM IN EPILEPSY

### 2.1 Metabolism and Homeostasis of GPLs

#### 2.1.1 Synthesis and Degradation of GPLs

 The synthesis of GPLs primarily occurs through the Kennedy and CDP-diacylglycerol (CDP-DAG) pathways. PCs and PEs are synthesized mainly *via* the Kennedy pathway, whereas PIs, PGs, and CLs are synthesized mainly *via* the CDP-DAG pathway [25, 26]. Phosphatidic acid (PA), an essential intermediate in all GPL synthesis, is primarily derived from glucose. In the Kennedy pathway, PA undergoes dephosphorylation to generate the DAG intermediate, whereas, in the CDP-DAG pathway, PA combines with CTP to form CDP-DAG. The Kennedy pathway steps are: choline and ethanolamine are phosphorylated by kinases to generate phosphocholine (Choline-P) and phosphoethanolamine (Ethanolamine-P). This process is catalyzed by the enzymes CTP: cytidylyltransferase (CCT) and CTP: cytidylyltransferase (ECT), which facilitate the formation of CTP-choline and CTP-ethanolamine, respectively. Finally, both are transferred to DAG under the catalysis of transferases to form PCs and PEs. PEs can also undergo methylation, catalyzed by phosphatidylethanolamine N-methyltransferase (PEMT), to form PCs. In the CDP-DAG pathway, myo-inositol, glycerol, or PGs are condensed with CDP-DAG, catalyzed by synthases, to generate PIs, PGs, or CLs, respectively [25-28]. In addition, the biosynthesis of PSs is also related to the Kennedy pathway. PSs are produced from PCs and PEs through the action of phosphatidylserine synthase (PSS). PSs can also be decarboxylated to form PEs within the inner membranes of mitochondria. This reaction is catalyzed by phosphatidylserine decarboxylase (PSD) (Fig. **1A**) [29, 30].

Various phospholipases mediate the degradation of GPLs. Phospholipase A (PLA) releases fatty acids from GPLs, producing lysophospholipids and free fatty acids. Lysophospholipids can be reacylated into new GPLs. Phospholipase C (PLC) hydrolyzes the bond between phosphate and glycerol, yielding DAG and a phosphorylated head group. Phospholipase D (PLD) hydrolyzes the bond between phosphate and the head group, forming PA and a free head group (Fig. **1B**) [4].

#### 2.1.2 Possible Mechanisms for Maintaining GPL Homeostasis

Maintaining GPL homeostasis relies on the precise coordination of biosynthesis and degradation. For example, CCT overexpression accelerated PC synthesis, but their levels did not increase because of the simultaneous acceleration of PC degradation [31]. Similarly, PSS1 overexpression increased PS synthesis, but intracellular PS levels remained unchanged due to accelerated PS degradation and decarboxylation [32]. Furthermore, in PSS2 knockout mice hepatocytes, PS levels were normal due to reduced PS degradation [33]. Human study indicates that increased PEMT expression in colon adenocarcinoma reduces PCs synthesized *via* the Kennedy pathway while increasing those produced through PEs methylation [34]. Similarly, elevated PEMT expression in non-small-cell lung cancer is associated with poor prognosis [35]. Moreover, the SNP rs7949 in the PEMT gene has been linked to a higher risk of Alzheimer’s disease [36]. In addition, PSS2 expression in the cerebral cortex of patients with major depressive disorder is lower than in healthy individuals [37]. These findings highlight the essential role of coordinated GPL synthesis and degradation, emphasizing that dysregulation of key enzymes is closely associated with disease pathogenesis. Acyl chain remodeling of GPLs (LANDs cycle) is also an essential mechanism for homeostasis maintenance, which involves PLA1 or PLA2-mediated removal of acyl chains and reacylation mediated by acyl transferases [38, 39]. This remodeling process ensures the specific molecular composition of GPLs and is involved in generating arachidonate cascade signaling and repairing OxPLs [3, 38, 40].

### 2.2 Metabolism Disorders of GPLs in Patients and Animal Models with Epilepsy

Disruptions in GPL metabolism have been documented in epilepsy research. Srivastava *et al*. [41] reported elevated levels of triglycerides, PLs, and cholesterol in the brain tissue of rats in a post-traumatic epilepsy (PTE) model, accompanied by aggravated lipid peroxidation. This indicates oxidative stress-induced lipid degradation. Kainate is often used to construct acute epileptic seizure models. After kainate was applied to hippocampal neurons, levels of GPLs containing polyunsaturated fatty acids decreased [42]. Similarly, GPL levels were reduced in the hypothalamus and plasma of acute seizure mice [43]. Another study [24] revealed that acute seizures led to elevated lipid levels in the dorsal hippocampus and reduced lipid levels in the ventral hippocampus of mice. This change in metabolism was particularly pronounced in PCs. Studies indicate that children with drug-resistant epilepsy exhibit lower plasma levels of PCs, PEs, and PIs compared to those with well-controlled epilepsy [19]. In human studies, positively charged GPLs (PCs and PEs) were significantly downregulated, and negatively charged GPLs (PSs and PGs) were upregulated in the hippocampal tissue of individuals with temporal lobe epilepsy, suggesting a transformation in GPL metabolism in the hippocampus of patients with epilepsy [44, 45]. Whether these metabolic changes represent a hallmark of generalized metabolic dysregulation in epilepsy or contribute directly to functional impairments remains uncertain, highlighting the need for further in-depth investigation.

### 2.3 Mechanism of GPL Disorder Caused by Epileptic Seizures

#### 2.3.1 Neuroinflammation Enhances PLA2 Activity

Neuroinflammation is an inflammation of neural tissue triggered by various endogenous or exogenous factors. It is characterized by peripheral immune cell recruitment, reactive neuroglia proliferation, and elevated levels of multiple inflammatory mediators [46]. Human PLA2s are expressed and secreted by macrophages, monocytes, T-lymphocytes, and neutrophils. Elevated levels of several PLA2 subtypes have been detected in the blood of patients with inflammatory and autoimmune diseases [47]. High PLA2 concentrations have been observed in the synovial fluid of patients with arthritis [48]. Elevated PLA2 levels have also been detected in the plasma of patients with pancreatitis, inflammatory bowel disease, and septic shock [49]. Research has demonstrated that the upregulation of key inflammatory mediators, including interleukin (IL)-1β, tumor necrosis factor (TNF)-α, cyclooxygenase (COX)-2, and C-X-C motif chemokine ligand following an epileptic seizure, can contribute to secondary brain damage and increase the risk of recurrent seizures [50]. PLA2 activity is increased under pathological conditions, and inflammatory cytokines such as TNF-α and IL-1 have been implicated. In addition, calcium ion (Ca^2+^) overload triggered by calcium ion (Ca^2+^) in-flow can also overstimulate PLA2, resulting in pathological catabolism of GPLs (Fig. **2**) [3, 51]. Ueda *et al*. [52] found elevated PLA2 levels during epileptic seizures in PTE rats. In another study, the application of pentylenetetrazol to induce epileptic seizures in rats revealed significantly higher concentrations of secretory PLA2, considerably lower levels of PCs in the hippocampus and cerebellum, and slightly higher levels of PEs in the cerebellum in the epilepsy group when compared with the control group [53]. In a study by Lerner *et al*. [24], PLA2 was elevated in both the dorsal and ventral hippocampus of the epilepsy mouse model. Despite the increased PLA2 activity in the hippocampus, its upregulation exhibited distinct effects on lipid metabolism in the two hippocampal subregions. PLA2-mediated destruction of neuronal membrane GPLs occurred only in the ventral hippocampus, characterized by reduced PCs, whereas GPL levels increased in the dorsal hippocampus despite elevated PLA2 expression. These findings suggest that subregions of the PLA2 family in the brain may be functionally specific, which may be one of the reasons for the variability in the metabolism characteristics of GPLs between different tissues or regions. Additionally, PLA2 plays a key role in the deacylation-reacylation cycle of PLs, contributing to the restoration of neuronal membrane integrity by balancing free polyunsaturated fatty acids, a critical mechanism for neuronal self-repair under pathological conditions [3].

#### 2.3.2 Oxidative Stress Causes Lipid Peroxidation

Reactive oxygen species (ROS) and reactive nitrogen species (RNS) are tightly regulated under physiological conditions and are involved in physiological processes like signal transduction, autophagy, apoptosis, and mitosis [54]. However, oxidative stress represents a biochemical state characterized by an imbalance between the production and degradation of ROS/RNS, resulting in damage to cell membranes, mitochondria, enzymes, and DNA, ultimately causing neuronal necrosis [54]. Brain tissue has limited amounts of reduced glutathione and lacks significant levels of catalase, glutathione peroxidase, or vitamin E, making neurons susceptible to damage from oxidative stress [55]. As an extremely lipid-rich organ, the brain is prone to lipid peroxidation, triggered by abnormal free radicals generated in pathological states. This oxidative damage predominantly affects GPLs in nerve membranes, leading to the destruction of GPL composition [56, 57]. The products of GPLs peroxidation are OXPLs, which are biomarkers of oxidative stress and contribute to the destruction of GPLs in nerve membranes (Fig. **2**) [58, 59]. Evidence supports the role of oxidative stress in seizures, with clinical studies primarily focusing on patients with drug-resistant epilepsy. Patients with progressive myoclonic epilepsy have reduced plasma superoxide dismutase (SOD) activity. High-dose N-acetylcysteine (NAC) pretreatment demonstrated general benefits in these patients [60]. Pre- and post-surgical measurements of SOD, catalase, and glutathione peroxidase activity, along with lipid and protein oxidation markers, revealed altered antioxidant enzyme activity in the preoperative stage and increased oxidative damage marker levels. Post-surgery, all research variables except SOD activity tended to normalize [61]. In a previous study, PL levels in the brain tissue of PTE rats were significantly elevated than those in healthy controls, positively correlating with lipid peroxidation levels [41]. In rats subjected to electrically induced epileptic seizures, lipid peroxidation levels in the brain were elevated [62, 63]. Similarly, in an epileptic seizure model constructed by experimental injection of pentylenetetrazol, increased lipid peroxidation levels were observed in the hippocampus, cortex, and striatum [64]. Malondialdehyde is a byproduct of lipid peroxidation, and serum malondialdehyde levels are reduced by oxcarbazepine, suggesting that mitigating lipid peroxidation may be one of the pharmacological mechanisms of oxcarbazepine [65]. In addition, neuronal membrane lipid peroxidation has been linked to various neurodegenerative diseases, such as Alzheimer’s disease [66], Parkinson's syndrome [67], and multiple sclerosis [68]. Epileptic seizures frequently occur during the middle to late stages of certain neurodegenerative diseases, indicating that epilepsy may be a neurodegenerative disease itself or a clinical manifestation of a stage of neurodegenerative diseases, sharing a similar pathologic basis [69, 70]. In conclusion, substantial experimental evidence indicates that oxidative stress plays a dual role in epilepsy, acting as both a potential trigger and a consequence of epileptic seizures. This bidirectional relationship underscores its critical involvement in the pathophysiology of epilepsy, highlighting the need for further research to clarify its mechanisms and therapeutic implications [71, 72].

### 2.4 Mechanisms of Epilepsy Caused by GPL Disorder

#### 2.4.1 Ion Channels Dysfunction

 PIs consist of phospho-1,2-DAG and a myo-inositol head group, which are synthesized in the endoplasmic reticulum. PIs play important roles in cellular signal transduction, ion channel regulation, endocytosis, exocytosis, and metabolic processes [73]. Physiologically, PIs can be phosphorylated by various kinases, resulting in multiple phosphorylated forms of PIs (PIPn). Among these, phosphatidylinositol-4,5-bisphosphate (PIP2) has received much attention [73, 74]. PIP2, a negatively charged lipid, plays a key role in regulating ion channels and thereby affects neuronal excitability. The KCNQ gene encodes the Kv7.2 and Kv7.3 subunits, which are responsible for a specific type of neuronal K^+^ current. Kv7 voltage-gated K^+^ channels generate currents only when membrane PIP2 levels reach a critical threshold, demonstrating an essential functional reliance on PIP2 levels. Research has demonstrated that mutations in the KCNQ gene can lead to early-onset neonatal epilepsy [75-77]. Soldovieri *et al*. [78] found that in children with epilepsy caused by mutations in genes related to Kv7.2 subunits, increasing PIP2 levels *via* PI(4)P5-kinase (PIP5K) partially restored Kv7.2 channel functionality. Furthermore, dynamic alterations in PIP2 levels can influence the voltage sensitivity of cortical KCNQ gene-related channels, impacting cortical excitability [79-81].

#### 2.4.2 Gene Mutations

Glycosylphosphatidylinositol (GPI)-anchored proteins represent a distinct category of lipid-linked neuronal membrane proteins, crucial for synaptic regulation, cytoskeletal remodeling, and signal transduction. They additionally serve as structural and signaling platforms essential for the localized regulation of neuronal function [82]. In mammalian cells, GPI synthesis involves the stepwise addition of monosaccharides, acyl chains, and phosphoethanolamine residues to PIs within the endoplasmic reticulum [83]. Mutations in genes responsible for GPI-anchored protein biosynthesis can impair their function, leading to epilepsy, developmental disorders, cognitive dysfunction, and ataxia [84]. Davids *et al*. [84] reported seven cases of ARV1 biallelic splice mutations from two different families. Affected infants exhibited epileptic encephalopathy, developmental delays, central hypotonia, and delayed speech development. The PIGH gene encodes a transferase that catalyzes the initial step of GPI-anchored protein biosynthesis. Additionally, PIGH homozygous mutation leads to epileptic encephalopathy and delayed myelination [85]. Mutations in the PIGT [86], PIGU [87], PIGW [88], PIGO [89], and PIGL [90] genes have also been reported to cause epilepsy by disrupting the activity of GPI-anchored proteins. Beyond GPI, the MBOAT7 gene encodes an O-acyltransferase domain comprising seven proteins. This domain represents one of four enzymes participating in the remodeling of PLs [91]. Yalnızoǧlu *et al*. [92] reported the clinical features of 12 patients with defects in the MBOAT7 gene, including early-onset epilepsy, developmental delays, and intellectual disabilities.

#### 2.4.3 Epileptogenic effect of AA and its Metabolites

Neuroinflammation may preexist in brain tissue as a cause of epilepsy, increasing susceptibility to epilepsy. Direct evidence indicates that individuals with autoimmune disorders exhibit a higher prevalence of epilepsy compared to that of the general population [93]. Similarly, glioblastoma is a common cause of epilepsy, and its tumor microenvironment releases large amounts of inflammation signals [94, 95]. Chronic neuroinflammation, such as microglia activation, is also observed in brain tissues of individuals diagnosed with focal cortical dysplasia (FCD) and tuberous sclerosis [96, 97]. The presence of neuroinflammation upregulates PLA2 activity, which breaks down GPLs. AA, an unsaturated fatty acid, is a component of GPLs, which is decomposed into free forms under PLA2 catalysis. Free AA lies at the core of a series of metabolic pathways collectively termed the arachidonate cascade, which serves as a critical source of inflammatory mediators and second messengers [98, 99]. However, the same signaling pathways also lead to excitatory brain damage when overstimulated. Disruptions in GPL homeostasis and signaling may serve as critical factors in the initiation and progression of epilepsy pathology (Fig. **2**). Research has demonstrated that AA can induce excitotoxicity by promoting glutamate release from neuroglia, reducing glutamate reuptake by neurons, enhancing glutamate receptor activity, and inhibiting K^+^ and Cl^-^ channels [100]. Porta *et al*. [101] reported that reduced serum AA levels correlate with the anticonvulsant effects of the ketogenic and modified Atkins diets. Free AA in hippocampal neurons of mice suppresses transient currents in inhibitory interneurons and broadens action potentials, indicating that AA may intensify the severity of epileptic seizures [102]. Furthermore, studies have demonstrated that reducing intracellular AA levels can alleviate the severity of epileptic seizures [103].

Free AA has three main metabolism pathways: prostaglandins and thromboxane A2 (TXA2), catalyzed by COX. Cysteinyl leukotrienes (CysLTs), hydroxyeicosatrienoic acids (HETEs), and lipoxins catalyzed by lipoxygenase (LOX). HETEs and epoxyeicosatrienoic acid isomers (EETs) catalyzed by cytochrome P450 (P450). COX exists in three isoforms: COX-1, COX-2, and COX-3, with the COX-2 metabolic pathway being particularly significant in the onset and progression of epilepsy (Fig. **2**) [100]. COX-2 expression is upregulated in the brains of patients with epilepsy and animal models, where the COX-2 pathway contributes to neuroinflammation, glutamate-mediated excitotoxicity, blood-brain barrier (BBB) destruction, and cognitive impairments [50, 104, 105]. In pilocarpine-induced SE mouse models, neuronal COX-2 exacerbated hippocampal inflammation [106]. Weidner *et al*. [107] detected elevated COX-2 expression in microglia from surgically resected brain tissue of temporal lobe epilepsy patients. Additionally, COX-2 upregulates the BBB transporter P-glycoprotein, impairing the delivery of ASMs to brain targets and potentially contributing to drug-resistant epilepsy [108]. COX-2-derived prostaglandins have been a major focus in epilepsy research, with prostaglandin E2 (PGE2) and its downstream EP2 receptors being the most extensively studied [109, 110]. For instance, elevated PGE2 levels have been observed in both the plasma and brain tissues of patients with epilepsy [111, 112]. PGE2 regulates postsynaptic membrane excitability and synaptic plasticity in hippocampal dentate granule neurons, and selective COX-2 inhibitors have been shown to significantly lower postsynaptic membrane excitability [113].

#### 2.4.4 Toxic effects of OxPLs

To date, few studies have explored the mechanisms by which OxPLs mediate epilepsy pathogenesis, but it has been found that the degree of lipid peroxidation is positively correlated with epilepsy severity [41]. Currently, OxPLs-mediated inflammatory response, apoptosis, cell necrosis, axonal degeneration, and neuronal damage have been demonstrated in select neurological diseases [16, 114-116]. Although the pathogenic mechanism of OxPLs in epilepsy is unknown, their role in other disorders may be suggestive. The E06 monoclonal antibody, which detects OxPLs, specifically binds to oxidized phosphatidylcholines (OxPCs) while lacking specificity for other OxPLs. Consequently, OxPCs are the most extensively recognized and characterized OxPLs [58, 59]. The EO6 antibody, which specifically targets OxPCs, binds to dying cells, indicating the presence of an OxPCs-specific epitope on their surface [117]. These OxPCs are classified as dangerous or damaged epitopes [118], recognized by macrophage scavenger receptor CD36, promoting phagocytosis and clearance by macrophages [119]. OxPLs also interact with CD14 on macrophage surfaces, triggering intracellular signaling cascades through Toll-like receptor 4 (TLR4). These cascades activate the transcription factor NF-kappaB (NF-kB), which promotes the transcription and release of inflammatory cytokines like IL-1β. This, in turn, hyperactivates macrophages, leading to tissue damage [120]. In mice, influenza virus infection promotes the formation of OxPLs, which hyperactivates lung macrophages and aggravates acute lung injury [121]. Another study showed that after bleomycin-induced lung injury, OxPLs accumulated in alveolar macrophages, leading to increased foam cell formation and aggravated lung fibrosis [122]. OxPLs actively contribute to β-amyloid peptide formation in Alzheimer's disease, highlighting their neurotoxicity in this condition [123]. Evidence indicates that lipid peroxidation is markedly elevated in dopaminergic neurons of Parkinson's disease patients, with the resulting OxPLs promoting α-synuclein formation [124, 125]. Moreover, deposition of OxPLs has been identified in spinal cord injury tissue [114]. Therefore, under the condition of insufficient clearance of necrotic cells, OxPLs will accumulate, over-activate macrophages, aggravate immune inflammatory reactions, and lead to tissue damage (Fig. **2**).

CLs are predominantly located in the mitochondrial inner membrane. Brain mitochondria have a variety of CL molecules, but they are easily affected by oxidation because of their polyunsaturated nature (Fig. **2**) [126]. For example, cytochrome c (Cyt-c), a protein in the mitochondria electron transport chain complex, is thought to selectively peroxidize polyunsaturated fatty acids in CLs [16]. Additionally, oxidative depletion of CLs and accumulation of over 150 new oxidized cardiolipin (OxCL) molecules were found in traumatic brain injury animal models. Notably, the application of targeted electron scavengers within mitochondria effectively prevented the oxidation of CLs in the brain and reduced neuronal death [126].

### 2.5 Therapy

#### 2.5.1 Control Neuroinflammation

The IL-1 receptor 1 (IL-1R1)-TLR4 axis and transforming growth factor-β (TGF-β) signaling pathways have been identified as contributors to epilepsy pathogenesis [127, 128]. Interventions in these two pathways have demonstrated clinical therapeutic effects in epilepsy and neurological injuries. Pathological signals commonly activate the IL-1R1-TLR4 signaling pathway. Specifically, TLR4 is activated by high mobility group box 1 protein (HMGB1), which is released from neurons. IL-1R1 activation occurs *via* IL-1β, a cytokine released by neurons, neuroglia, or macrophages. The release process is mediated by the catalytic subunit of caspase 1 [127, 129-131]. Evidence suggests that blocking IL-1R1, inhibiting caspase 1 to prevent IL-1β production, blocking TLR4, or inhibiting HMGB1 can reduce acute symptomatic seizures and spontaneous recurrent seizures in animals [132-134]. Anakinra is an IL receptor blocker used in pediatric patients diagnosed with febrile infection-related epilepsy syndrome, a disorder characterized by frequent seizures. Anakinra is also used to treat systemic inflammatory adolescent drug-resistant epilepsy related to IL-1 signaling, in which it significantly controls seizures unresponsive to steroid hormones or other ASMs [132, 135]. Additionally, delivering specific non-viral small interfering RNA (siRNA) into the cerebral ventricles of epileptic rats or rats with SE to target caspase 1 reduces the epileptic seizure incidence and duration and reduces neuronal damage [133, 136]. Evidence indicates that TGF-β signaling contributes to the pathological hyperexcitability of astrocytes [128]. Drugs that block TGF-β signal activation, such as losartan, an angiotensin receptor blocker, prevent astrocyte dysfunction and reduce epilepsy incidence and seizure frequency [137].

#### 2.5.2 Inhibition of PLA2 and COX-2

PLA2 inhibitors target the early biosynthesis of inflammatory mediators, protecting nerve cells from inflammatory damage. To successfully treat neuroinflammation, the ideal PLA2 inhibitor should be region-specific and capable of crossing the BBB to target the injury site [24]. PLA2 inhibitor has been proven to improve the impairment of membrane permeability, fluidity, and ion homeostasis caused by PLA2 stimulation [123, 124]. For example, PLA2 activity can be inhibited by CDP-choline, used to treat ischemic diseases and head injuries. Its function of inhibiting PLA2 activity reduces AA and ROS levels, contributing to the stability of neuronal membranes and protecting neurons from glutamate-mediated excitotoxicity [138, 139]. However, PLA2 inhibitors are currently mainly non-specific, underscoring the need to design and synthesize specific PLA2 inhibitors.

As COX-2 is implicated in amplifying inflammatory responses, it has become a primary target for drug-based interventions in epilepsy. Blocking the COX-2 cascade can prevent epilepsy occurrence and reduce chronic epileptic seizure frequency. Selective COX inhibitors like rofecoxib and celecoxib have been shown to raise the threshold for acute seizures in pentylenetetrazol-induced mice models [140, 141]. Celecoxib can also decrease the occurrence and intensity of chronic epileptic seizures after SE in rats [142]. Aspirin, a non-selective COX-1 and COX-2 inhibitor, has been used in pilocarpine-induced SE rat models to temporarily mitigate spontaneous recurrent seizures during chronic epilepsy while minimizing hippocampal neuron damage [143]. The NOD-like receptor 3 (NLRP3) inflammasome contributes to the pathogenesis of multiple central nervous system (CNS) diseases, including epilepsy. In rat models, ibuprofen inhibited COX-2, downregulated NLRP3, and its downstream IL-18, and exerted both anti-epileptic and neuroprotective effects [144]. Selective COX-2 inhibitors, such as celecoxib, prevent seizure-induced upregulation of P-glycoprotein in the BBB, thereby enhancing the delivery of ASMs to the brain. Consequently, COX-2 inhibition increases ASM concentrations at epileptic foci, improving treatment efficacy in drug-resistant epilepsy [145]. In addition, the application of a new EP2 antagonist in mice with SE reduced mortality, accelerated the recovery of body weight, improved cognitive function, and mitigated damage to the BBB and hippocampal neurons [146]. Although COX-2 inhibitors have been extensively studied for epilepsy treatment, clinical trials remain limited due to their high risk of adverse effects. Sturge-Weber syndrome is a rare neurological disease. A study on patients treated with aspirin reported seizure reduction in 21 of 34 cases [147]. Lance *et al*. [148] further confirmed these findings, reporting seizure control in 91% of patients receiving low-dose aspirin.

#### 2.5.3 Neuroprotective effect of eCBs

PLs in the brain have a different metabolic pathway as the precursor of eCBs [149]. The most characteristic eCBs include anandamide (AEA) and 2-arachidonoylglycerol (2-AG) [149]. There is a metabolic link between eCBs and the arachidonate cascade, as AEA and 2-AG can be hydrolyzed to AA by fatty acid amide hydrolase (FAAH) and monoacylglycerol lipase (MAGL), respectively (Fig. **2**) [150]. Research indicates that eCBs exhibit both anti-inflammatory and neuroprotective properties. By inhibiting their degradation, promoting their synthesis, or increasing receptor density, eCBs effectively control epileptic seizures and reduce glutamate-mediated excitotoxicity [151]. AEA levels in the cerebrospinal fluid of patients with temporal lobe epilepsy and the amygdala of epileptic rats are reduced. AEA exhibits not only antiepileptic effects but also relieves comorbid emotional dysfunction associated with epilepsy [152, 153]. Colangeli *et al*. [154] found that inhibiting FAAH aggravates synaptic damage caused by epileptic seizures. This may represent a potential therapeutic strategy for controlling epileptic seizures and improving memory impairment associated with epilepsy by stimulating FAAH. In addition, injecting a selective and irreversible MAGL inhibitor can alleviate the neuronal damage and cognitive dysfunction caused by SE in mice with diazepam resistance and decrease IL-1β and COX-2 expression [155].

#### 2.5.4 Antioxidant and OxPLs Clearance

Substantial evidence supports the role of oxidative stress in epilepsy pathophysiology and neuronal death, suggesting that antioxidants may provide neuroprotection in epilepsy patients. In pilocarpine-induced seizure models, ascorbic acid exhibited neuroprotective and anticonvulsant effects, potentially due to its ability to reduce lipid peroxidation and increase catalase activity [156]. α-Tocopherol's neuroprotective and anti-epileptic properties in humans and animal models may stem from its ability to reduce oxidative stress and inhibit neuroinflammation [157]. Laboratory and clinical studies indicate that melatonin exerts anticonvulsant effects by scavenging hydroxyl radicals, boosting glutathione peroxidase activity, and reducing ROS production and lipid peroxidation [158]. The combination of sulforaphane and NAC effectively controls spontaneous seizures and preserves hippocampal neurons in SE rat models. Treated animals exhibited significant cognitive improvement [159]. Clinical data on the use of antioxidants in epilepsy patients remain scarce. A randomized, double-blind, placebo-controlled trial evaluated the effect of alpha-tocopherol supplementation alongside ASMs in 24 children with drug-resistant epilepsy, reporting significant seizure reduction in 10 of 12 cases [160]. However, another study found no significant change in seizure frequency among 43 drug-resistant epilepsy patients receiving vitamin E treatment [161]. Yürekli *et al*. [162] reported that co-administration of topiramate and selenium enhanced plasma total antioxidant status and glutathione peroxidase levels in drug-resistant epilepsy patients. A recent clinical trial assessed the effect of adjunct melatonin therapy in generalized epilepsy treatment. The experimental group receiving melatonin combined with valproic acid achieved a higher seizure freedom rate than the control group [163].

Given that OxPLs are involved in important pathological activities in the body, exploring whether inactivation or removal of OxPLs can be used for disease treatment is important. Research has suggested that high-density lipoprotein (HDL) may serve as a potential treatment for atherosclerosis. HDL mediates OxPL degradation *via* platelet-activating factor acetylhydrolase (PAF-AH) and lecithin-cholesterol acyltransferase (LCAT) [164, 165] and counteracts OxPL toxic effects by reducing cell sensitivity [166]. Thus, promoting the inactivation of OxPLs may be a potential mechanism by which HDL exerts anti-atherosclerotic and anti-inflammatory effects. Microglia neutralize OxPCs that accumulate at lesion sites [68]. Microglia in the lesion site of OxPCs upregulate triggering receptor expressed on myeloid cells 2 (TREM2). TREM2 is a lipid-sensitive receptor that plays a role in clearing myelin cholesterol [167, 168]. *In vitro*, recombinant TREM2 binds to purified OxPCs. In TREM2-deficient mice, the neurodegeneration mediated by OxPCs is more severe, indicating that the up-regulation of TREM2 is the mechanism of microglia clearing OxPCs [68]. Therefore, enhancing microglia-mediated OxPC clearance may be a possible therapeutic approach [169]. Additionally, EO6 is an antibody that can neutralize OxPCs. In mice, EO6 reduced OxPCs-mediated neurodegeneration. However, considering that the antibody is injected into the CNS parenchyma through stereotaxic techniques, it is uncertain whether peripheral injection of EO6 can neutralize OxPCs [68].

#### 2.5.5 Regulate PIP2 Levels

KCNQ channels are key structures that regulate the excitability of neurons, and PIP2 levels strictly regulate the maintenance of their physiological function. Protein arginine methyltransferase 1 (Prmt1) modulates KCNQ channel activity through methylation, helping to prevent neuronal hyperexcitability. The methylation of four arginine residues in the KCNQ2 channel by Prmt1 enhances the binding of KCNQ2 channels to PIP2. In contrast, the absence of Prmt1 reduces the affinity of KCNQ2 channels for PIP2, leading to a decline in channel activity [170]. Exogenous PIP2 supplementation has been shown to restore KCNQ current and normal excitability of neurons in mice lacking KCNQ activity [170]. Therefore, future research should further explore the mechanism of PIP2 in epilepsy pathogenesis and treatment and explore approaches to regulate PIP2 levels to control epilepsy pathogenesis.

## CONCLUSION

The synthesis and degradation of GPLs primarily occur in the endoplasmic reticulum and mitochondria, a sophisticated and complex process involving the participation of multiple enzymes. Maintaining GPL homeostasis in mammalian cells is a demanding process, necessitating precise coordination across synthesis, remodeling, degradation, and intracellular transport [4]. Although CCT is acknowledged as a critical enzyme in PC synthesis, the regulatory mechanisms of many other enzymes associated with GPL metabolism remain poorly understood. Similarly, the metabolism and transport processes of several intermediates are not fully elucidated. Difficulties in the purification and reconstitution of membranes and enzymes further hinder comprehensive insights into GPL metabolism [4, 171, 172]. Enzymes are essential for GPL interconversion and homeostasis and are implicated in various diseases. The overexpression of PEMT, PSS1, PSS2, and PSD has been linked to tumors [34, 35, 173], Alzheimer’s disease [36], metabolic disorders [174], and cardiovascular disease [175]. Thus, enzyme inhibitors hold promise as potential therapeutic agents. Currently, research on PEMT inhibitors remains limited, while PSS1 and PSD inhibitors have been developed but remain in the preclinical stage without clinical validation [176]. For instance, DS07551382, DS55980254, and DS68591889 have been identified as PSS1 inhibitors. *In vivo* and *in vitro* studies indicate that these inhibitors induce apoptosis in B-cell lymphoma cells [177, 178]. Thus, further research and development of GPL metabolic enzyme inhibitors are needed, followed by clinical trials contingent on their demonstrated safety and efficacy in laboratory studies. Future advancements may enable disease treatment through GPL metabolism regulation. Additionally, acyl transferases facilitate the reacylation of distinct fatty acid chains, generating GPL diversity in cell membranes and contributing to cellular functional specificity. Future studies should further investigate the mechanisms underlying GPL diversity in cell membranes and its functional significance [6].

Neuroinflammation not only plays a critical role in epilepsy occurrence but is also active during acute symptomatic seizures and chronic epilepsy [146, 147]. For instance, increased PLA2 activity disrupts calcium ion (Ca^2+^) homeostasis and degrades membrane GPLs, resulting in the production and accumulation of large amounts of AA and the destruction of nerve membranes [179-182]. Additionally, PLA2 can activate inflammatory cells, stimulating macrophages, neutrophils, eosinophils, monocytes, and endothelial cells to release inflammatory mediators [183]. Animal studies have demonstrated elevated PLA2 levels in the brains of epilepsy patients. However, human studies on this topic remain limited. Although some studies suggest that epilepsy patients with psychiatric symptoms exhibit elevated PLA2 levels, further research is needed to elucidate the role of PLA2 subtypes in epilepsy pathogenesis. Magnetic resonance spectroscopy is a non-invasive tool for metabolic analysis. Future clinical trials may utilize it to assess PLA2 level variations across different brain regions in epilepsy patients. Further investigations in animal and cell models can clarify PLA2’s role in epilepsy pathogenesis [24, 43, 181, 184]. Moreover, drugs that preserve GPLs and stabilize nerve membranes may be crucial in neuroprotection. PLA2 catalysis represents a rate-limiting step in the arachidonate cascade. Thus, PLA2 inhibition can suppress oxidative and inflammatory responses at the injury site. Ideal PLA2 inhibitors should be biocompatible, cell-permeable, and region-specific. Thus, developing BBB-permeable PLA2 inhibitors with minimal side effects may offer a novel therapeutic strategy for epilepsy [53, 182, 185]. AA and its metabolites modulate diverse cellular processes through interaction with receptors in various tissues, playing a pivotal role in the pathophysiology of neurological disorders, including epilepsy. While most studies suggest that AA promotes seizure activity, some suggest a potential protective role. Fraser *et al*. [186] suggested that the elevation of serum AA might result from the mechanisms underlying the seizure-suppressing effects of the ketogenic diet. Another study proposed that AA might serve as an endogenous protective factor against glutamate-induced excitotoxicity in drosophila [187]. However, the findings of these studies may be influenced by small sample sizes and the use of non-mammalian models. Notably, COX-2, a central regulator of early inflammatory responses, significantly contributes to brain hyperexcitability and inflammation both prior to and following epileptic seizures. The efficacy of non-steroidal anti-inflammatory drugs (NSAIDs) in experimental epilepsy models has been documented. Although NSAIDs exhibit well-established anti-inflammatory effects, prolonged use is associated with an increased risk of adverse effects. More importantly, while these drugs have demonstrated efficacy in animal epilepsy models, their effects remain complex and insufficiently evaluated in large-scale clinical studies. Currently, no selective COX-2 inhibitors have been unequivocally shown to effectively control epilepsy with minimal side effects. Future research should focus on rigorously evaluating the safety and therapeutic efficacy of COX-2 inhibitors in epilepsy patients through well-designed clinical trials [188].

Growing evidence highlights the significance of oxidative stress in CNS disorders. Epilepsy is associated with redox system imbalance, suggesting that antioxidant therapy may serve as a potential adjunctive treatment. However, the clinical efficacy of antioxidant therapy in epilepsy remains inconclusive. NAC may exert neuroprotective effects by slowing neuronal degeneration. Further clinical investigations are required to evaluate NAC’s efficacy in drug-resistant epilepsy patients [60]. Vitamin C, vitamin E, glutathione, and melatonin are key components of endogenous antioxidant defense mechanisms. Given the limited research available, the efficacy and safety of natural antioxidant therapies for CNS disorders remain unclear [156, 157, 189]. For drug-resistant epilepsy patients, current treatment options primarily include surgery and a ketogenic diet. However, drug-resistant epilepsy remains a major clinical challenge. Currently, no definitive evidence supports the use of antioxidants as adjunctive therapies for drug-resistant epilepsy. Thus, further investigation into the underlying mechanisms of drug-resistant epilepsy is crucial for developing novel therapeutic strategies to enhance patient quality of life [190]. OxPLs exert their pathological effects primarily by modulating inflammatory responses. OxPLs are well-characterized inflammatory modulators in atherosclerosis [191], and emerging evidence suggests their involvement in other diseases as well [121-124]. Currently, the mechanisms underlying OxPL involvement in epilepsy remain poorly understood. For instance, OxPLs have been shown to interact with Toll-like receptors, raising the possibility that this interaction contributes to epilepsy pathogenesis [192]. OxPCs are the most extensively studied OxPLs, and a key research objective is to elucidate their role in epilepsy. However, several challenges remain. Although the E06 antibody selectively binds OxPCs, it lacks the specificity to distinguish OxPC subtypes. Thus, lipidomics may facilitate the comprehensive analysis of OxPC profiles in the CNS of epilepsy patients [193]. From a therapeutic standpoint, no effective CNS-penetrating therapies currently exist to neutralize OxPLs or mitigate their neurotoxic effects. An optimal neutralizing antibody should achieve sufficient BBB penetration following peripheral administration [68]. Furthermore, enhancing microglia-mediated OxPL clearance may represent a promising therapeutic approach [169].

PIP2 is critical for the normal functioning of certain K^+^ channels and is closely related to epilepsy occurrence. Future research should continue to explore the potential links between ion channel gene mutations, PIP2 level regulation, and epilepsy, with the goal of developing new treatment strategies for ion channel-related epilepsy [81]. In addition, no effective treatment currently exists for the dysfunction of GPI-anchored proteins caused by genetic mutations. However, with the in-depth study of related genes and their functions, it will become possible to predict and treat diseases based on gene mutations. In summary, future efforts should focus on developing targeted therapies for genetic mutations to mitigate mutation-induced neuronal hyperexcitability and promote neuronal repair.

GPLs are indispensable biomolecules for brain function, and their metabolic dysregulation is strongly associated with epilepsy. A comprehensive understanding of GPL metabolism in epilepsy will provide critical insights into its molecular mechanisms and facilitate the identification of therapeutic targets. While numerous studies have established a strong link between abnormal GPL metabolism and epilepsy, current research primarily focuses on inflammation, oxidative stress, ion channel dysfunction, and gene mutation, suggesting potential therapeutic targets within these pathways. However, translating these findings into effective clinical treatments remains challenging. Although many drugs have demonstrated efficacy in laboratory settings, their safety and effectiveness in human epilepsy patients have not yet been fully established. Therefore, clinical translation should proceed in stages, guided by rigorous validation of safety and efficacy through animal models and cellular studies.

## Figures and Tables

**Fig. (1):**
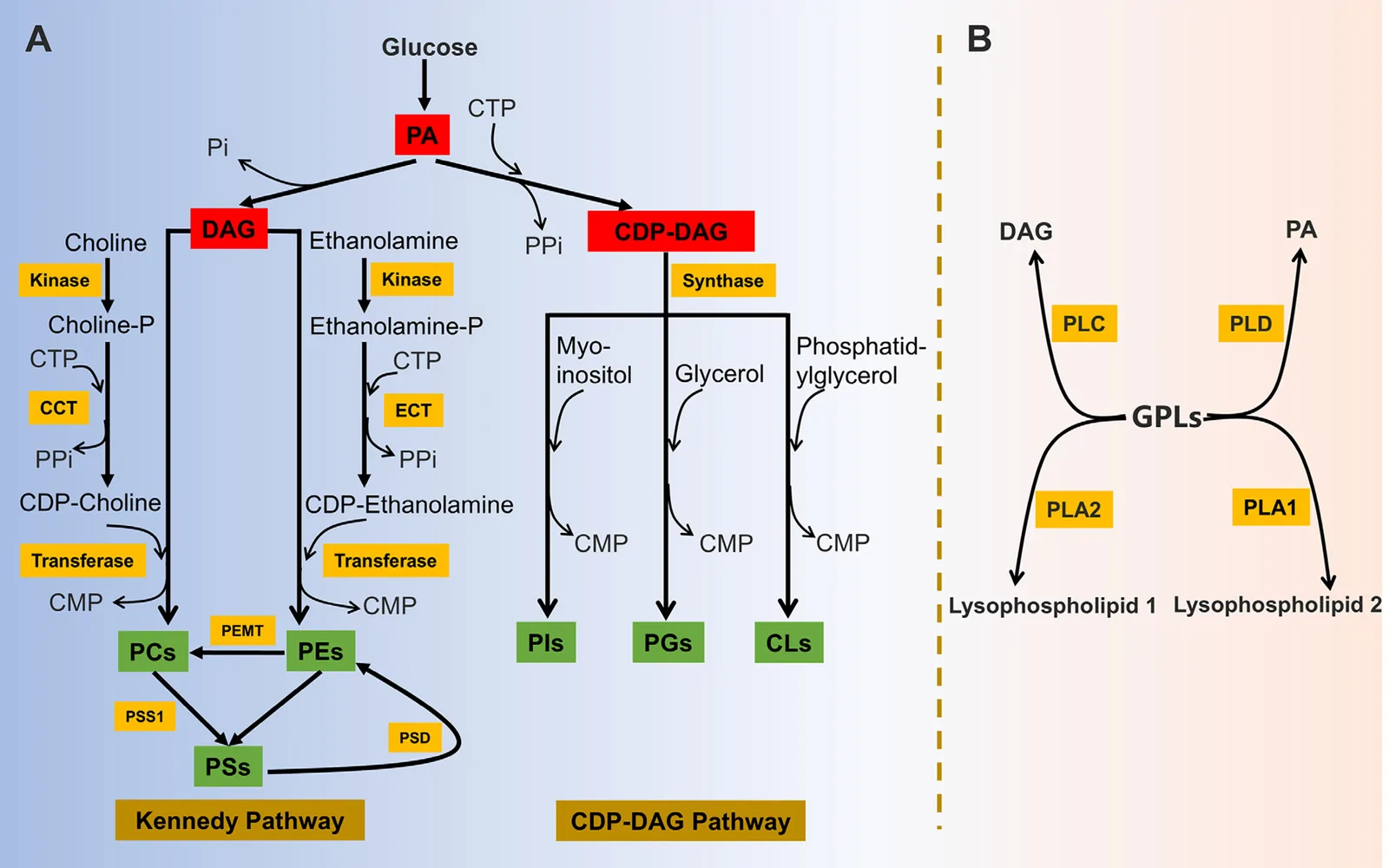
Synthesis and degradation of GPLs. (**A**) Synthesis of GPLs: PA, primarily derived from glucose, serves as a precursor in two pathways. Kennedy pathway: PA is dephosphorylated to DAG, which undergoes three enzymatic steps: (1) Kinase phosphorylates choline or ethanolamine to form Choline-P or Ethanolamine-P. (2) CCT and ECT catalyze the condensation of Choline-P/Ethanolamine-P with CTP, forming CTP-choline or CTP-ethanolamine. (3) Transferase transfers CDP-choline or CDP-ethanolamine to DAG, producing PCs or PEs. CDP-DAG pathway: CDP-DAG condenses with myo-inositol, glycerol, or phosphatidylglycerol to generate PIs, PGs, or CLs. Interconversion: PSS1 and PSS2 convert PCs and PEs into PSs, which can regenerate PEs *via* PSD. PEs also convert to PCs through PEMT catalysis. (**B**) Degradation of GPLs: PLA1 & PLA2: Remove fatty acids at sn-1 and sn-2, generating lysophospholipids. PLC: Cleaves phosphate-glycerol bonds, producing DAG. PLD: Hydrolyzes phosphate-head group bonds, yielding PA.

**Fig. (2):**
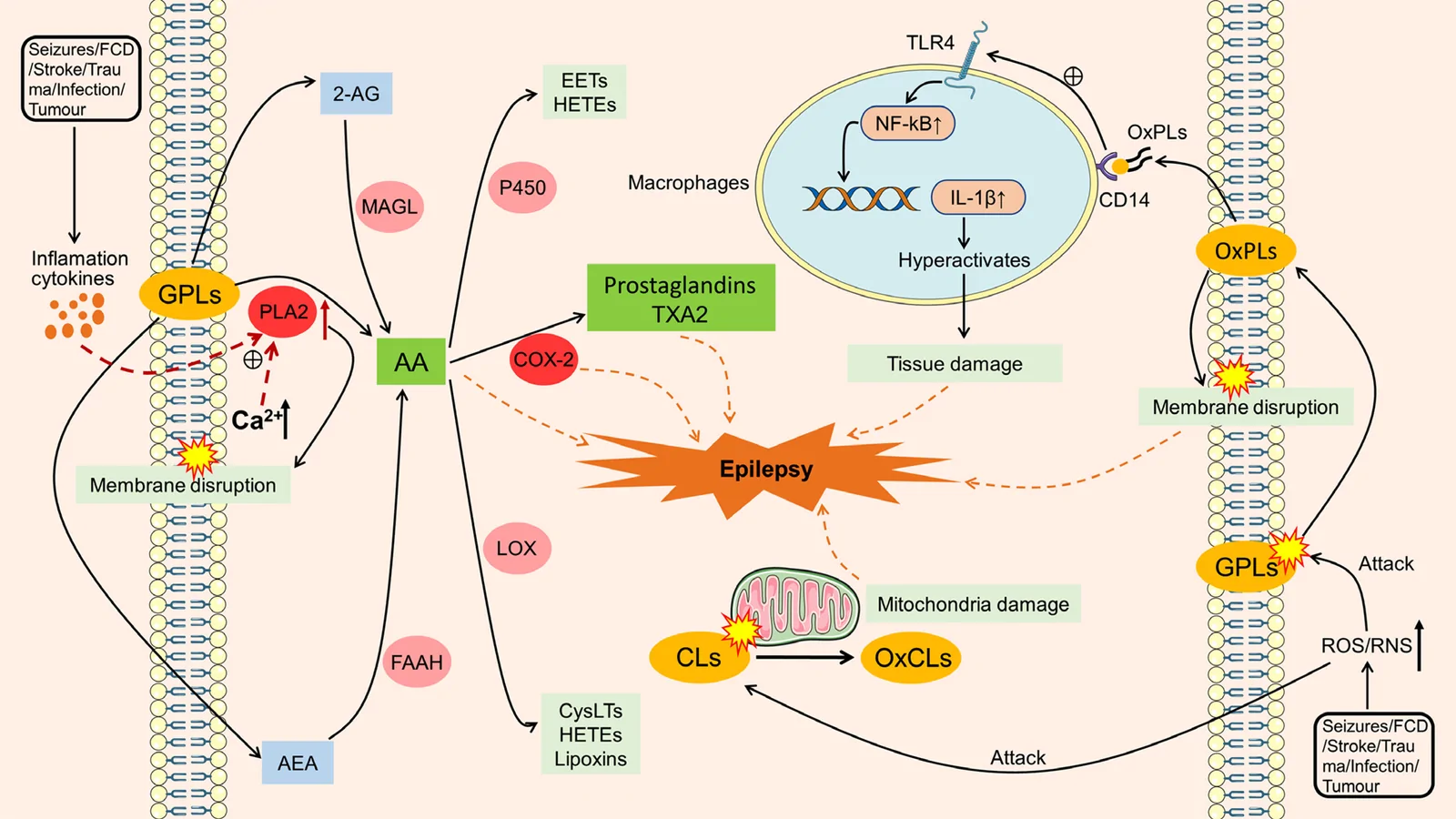
Potential mechanism linking cell membrane GPLs damage and epilepsy. Seizures, FCD, stroke, trauma, infection, and tumors activate inflammatory responses and oxidative stress, contributing to GPL damage and epilepsy. Inflammatory cytokines and calcium ions enhance PLA2 activity, leading to GPL breakdown, membrane damage, and the release of free AA. AA is further metabolized by P450, COX-2, and LOX to generate bioactive lipids, including EETs, prostaglandins, TXA_2_, CysLTs, HETEs, and lipoxins. Additionally, GPLs generate 2-AG and AEA, which are metabolized by MAGL and FAAH to produce free AA, further amplifying lipid signaling. Concurrently, oxidative stress induced by elevated ROS and RNS leads to the oxidation of GPLs and CLs, forming OxPLs and OxCLs, which compromise membrane integrity and mitochondrial function. OxPLs bind to CD14 on macrophages, triggering TLR4-mediated signaling cascades that elevate inflammatory cytokine levels, resulting in macrophage hyperactivation and subsequent tissue damage.

## LIST OF ABBREVIATIONS

AA: Arachidonic Acid
AEA: Anandamide
ASMs: Anti-seizure Medications
2-AG: 2-arachidonoylglycerol
BBB: Blood-brain Barrier
CCT: CTP: Phosphocholine cytidylyltransferase
CDP-DAG: CDP-diacylglycerol
CLs: Cardiolipins
CNS: Central Nervous System
COX: Cyclooxygenase
CysLTs: Cysteinyl Leukotrienes
DAG: Diacylglycerol
eCBs: Endocannabinoids
ECT: CTP: Phosphoethanolamine cytidylyltransferase
EETs: Epoxyeicosatrienoic Acid Isomers
FAAH: Fatty Acid Amide Hydrolase
FCD: Focal Cortical Dysplasia
GPI: Glycosylphosphatidylinositol
GPLs: Glycerophospholipids
HETEs: Hydroxyeicosatrienoic Acids
HMGB1: High Mobility Group Box 1 Protein
IL: Interleukin
LOX: Lipoxygenase
MAGL: Monoacylglycerol Lipase
NAC: N-acetylcysteine
NF-kB: NF-kappaB
NSAIDs: Non-steroidal Anti-inflammatory Drugs
OxCLs: Oxidized Cardiolipins
OxPCs: Oxidized Phosphatidylcholines
OxPLs: Oxidized Phospholipids
PA: Phosphatidic Acid
PCs: Phosphatidylcholines
PEs: Phosphatidylethanolamines
PEMT: Phosphatidylethanolamine N-methyltransferase
PGE2: Prostaglandin E2
PGs: Phosphatidylglycerols
PIP2: Phosphatidylinositol-4,5-bisphosphate
PIs: Phosphatidylinositols
PLA: Phospholipase A
PLC: Phospholipase C
PLD: Phospholipase D
PLs: Phospholipids
Prmt1: Protein Arginine Methyltransferase 1
PSD: Phosphatidylserine Decarboxylase
PSs: Phosphatidylserines
PSS1: Phosphatidylserine Synthase 1
PSS2: Phosphatidylserine Synthase 2
PTE: Post-traumatic Epilepsy
P450: Cytochrome P450
RNS: Reactive Nitrogen Species
ROS: Reactive Oxygen Species
SE: Status Epilepticus
TGF-β: Transforming Growth Factor-β
TLR4: Toll-like Receptor 4
TNF: Tumor Necrosis Factor
TREM2: Triggering Receptor Expressed on Myeloid Cells 2
TXA2: Thromboxane A2
